# The Accuracy of Transcutaneous Bilirubin as a Screening Test in Preterm Infants

**DOI:** 10.7759/cureus.42793

**Published:** 2023-08-01

**Authors:** Yunfai Ng, Timothy Maul, Sreekanth Viswanathan, Caroline Chua

**Affiliations:** 1 College of Medicine, University of Central Florida College of Medicine, Orlando, USA; 2 Emergency Medicine, Mount Sinai Hospital, New York, USA; 3 Cardiac Center, Nemours Children's Health, Orlando, USA; 4 Department of Bioengineering, University of Pittsburgh, Pittsburgh, USA; 5 Division of Neonatology, Nemours Children's Health, Orlando, USA; 6 Department of Pediatrics, University of Central Florida College of Medicine, Orlando, USA

**Keywords:** diazo method, phototherapy, bilirubinometry, neonatal jaundice, hyperbilirubinemia

## Abstract

Objective: To determine the accuracy of transcutaneous bilirubin (TcB) to predict total serum bilirubin (TSB) in preterm infants across gestational age (GA) ranges and to calculate the cost-effectiveness of TcB as the primary screening test of choice for neonatal jaundice in neonatal intensive care unit (NICU) settings.

Methods: Single-center retrospective study of infants aged ≤ seven days admitted to the NICU over a six-month period with a paired TSB and TcB, with or without phototherapy as part of their routine clinical care. Infants were divided into GA-specific groups as term, late preterm, moderate preterm, and very preterm. Measurement bias (bias=TSB-TcB) was calculated on the paired TSB and TcB values, and a Bland-Altman analysis was carried out. The impacts of additional infant-specific variables on the bias were assessed with univariate and multivariate linear regression techniques. The potential direct cost savings associated with the use of TcB as the primary screening test were calculated.

Results: A total of 263 paired TSB and TcB samples from 95 patients were included (130 paired samples from term (n=60), 75 from late preterm (n=21), 27 from moderate preterm (n=7), and 31 from very preterm (n=7)). The mean paired measurement bias across all the GA groups was -0.9 ± 2.9 mg/dL. The sensitivity and specificity of TcB in GA < 35 weeks were 92% and 62%, respectively. A conservative estimate of a one-third reduction in TSB measurement by using TcB as the primary screening test will have a direct cost saving of $3,148 over a six-month period.

Conclusion: Our data suggest that TcB is a safe and potentially cost-effective screening test for jaundice across GA groups.

## Introduction

Neonatal jaundice resulting from hyperbilirubinemia is common in infants admitted to neonatal intensive care units (NICU). About 50% of term and 80% of preterm infants develop jaundice in the first weeks of life, and about 10% of breastfed babies remain jaundiced at one month of age [1,2]. Because of the risk of bilirubin neurotoxicity, frequent measurement of bilirubin levels is a standard practice in NICUs. Jaundice severity is estimated based on hour-specific bilirubin levels and additional risk factors of each infant, and appropriate treatment decisions are made including optimizing hydration, the need for phototherapy, and exchange transfusion [3-4].

Currently, the total serum bilirubin (TSB) assay is the gold standard for the diagnosis and monitoring of jaundice in NICU infants, which requires repeated blood sampling. This is not only costly, time-consuming, and traumatic but also may contribute to iatrogenic anemia and increase the risk of infection, especially in preterm infants [5-7]. Transcutaneous bilirubin (TcB) is increasingly utilized as a cost-saving, convenient, and non-invasive alternative for screening and measuring total bilirubin levels [7-10]. TcB based on the reflectance photometry or transcutaneous colorimetry principle using a hand-held device, has been shown in various studies to be a fairly accurate estimate of TSB in term and near-term infants regardless of skin color [8-10].

In 2004, the American Academy of Pediatrics (AAP) called for additional studies on the cost-effectiveness and reproducibility of transcutaneous measurements of TSB, particularly in preterm and ill infants [11]. Recent studies on the use of TcB in preterm infants with or without phototherapy have shown reasonable accuracy in predicting TSB [5,6,12-17]. However, routine use and adoption have not been universal due to concerns about its accuracy in correlating with serum bilirubin levels across the gestational age (GA) range [5-8]. Considering the advantages associated with the use of TcB, a comparison study with TSB was planned before adopting it as the initial screening test of choice in our NICU’s routine clinical practice. We hypothesized that TcB would be a reliable and accurate screening test for infants with jaundice in a NICU setting.

Our study objectives were to determine the accuracy of TcB assays to predict TSB in term and preterm infants across the GA groups and to calculate the potential cost-effectiveness of TcB as the primary screening test of choice for neonatal jaundice in a NICU setting.

## Materials and methods

Patient selection

This single-center retrospective study was conducted at the all-referral tertiary NICU at Nemours Children’s Hospital, Florida, Orlando, Florida. Nemours Children’s Health Institutional Review Board approval was obtained prior to data collection. All infants aged ≤ seven days admitted to the NICU from December 2017 to May 2018 (6 months) with a paired TSB and TcB, with or without phototherapy as part of their routine clinical care, were included in the study. Patients who required exchange transfusion or whose paired sample exceeded one hour were excluded. Patient demographics, including GA, birth weight, sex, and race, were collected. Preterm infants were divided into GA-specific groups as follows: late preterm as 34 weeks to < 37 weeks, moderate preterm as 32 weeks to < 34 weeks, and very preterm as < 32 weeks. Clinical parameters at the time of the paired TcB and TSB measurements, such as feeding method, age, and phototherapy treatment, were also collected.

TcB measurement

The use of TcB was introduced to our NICU in 2017 as part of a quality improvement project to implement a screening protocol for hyperbilirubinemia in neonates using point-of-care technology. A TcB measurement was carried out by a trained NICU bedside-registered nurse (RN) within one hour of TSB measurement using a commercially available noninvasive bilirubin analyzer (BiliChek®, Philips, USA) [18]. Per manufacturer specifications, this FDA-approved device is intended for use in postnatal infants who are < 20 days old, weighing 2.1-11.1 pounds, and within the GA of 27-42 weeks pre-, during, and post-phototherapy [18]. Using spectral reflectance, multiple wavelengths were measured of varying components of the skin (dermal maturity, melanin, bilirubin, hemoglobin, etc.), and all interfering factors, but the bilirubin, were filtered out to offer a more objective measurement of skin differences in skin pigments than visual inspection [9]. The fiber-optic probe is calibrated using a disposable plastic tip (BiliCal, Philips, USA) prior to each measurement per manufacturer recommendations.

Following electronic calibration, the device is placed in contact with the forehead skin of an infant placed in a supine or cradled position. A small amount of pressure is applied over 1-3 seconds, and five readings are performed to average one numerical TcB measurement calculated in umol/L or mg/dL. For infants undergoing phototherapy, a patch of skin on the forehead was shielded during and after phototherapy using the recommended BilElipse® phototherapy patch for consistency. Current institutional guidelines follow the suggested use of phototherapy from Maisels et al. for preterm infants less than 35 weeks gestation [4], and for infants > 35 weeks, the AAP guidelines on hyperbilirubinemia are followed [3].

TSB measurement

TSB measurements were obtained through a sampling of capillary, venous, or arterial blood drawn by the NICU bedside nurse and sent to the Nemours Children’s Hospital laboratory for measurement. The TSB was measured by van den Bergh or “Diazo” reaction using the VITROS TBIL Slides and the VITROS Chemistry Products Calibrator Kit 4 on the VITROS 5600 Integrated System.

Cost-effectiveness

The hospital charges associated with TSB were ascertained from the hospital laboratory charge files and coding database. Indirect costs not measured included infants’ pain from the needle sticks, anemia from blood loss, and nursing time related to blood sample collection.

Statistical analysis

The TSB and TcB measurement biases were analyzed using paired t-test and Mann-Whitney U, as appropriate. Test bias was computed as the difference between TSB and TcB (bias=TSB-TcB). A Bland-Altman analysis was carried out on the paired TSB and TcB values, with variability defined as + 1.96 standard deviations of the mean bias, and the percentage of values that lay outside these limits of agreement indicating how well the two measures agree with each other over the range of clinical values were measured [19]. The impacts of additional factors on the bias were assessed with univariate (ANOVA or Kruskal-Wallis) and multivariate linear regression techniques. Covariates included GA as a continuous variable and race (Caucasian, African-American, or other), blood type (A, B, AB, O), feeding type (IV, breastmilk, formula, mixture), and the presence of phototherapy as categorical variables. Prior to model generation, outliers for the bias (more than 1.5 times the IQR below the 25th or above the 75th quartiles) were removed. A stepwise entry model was utilized. Model assumptions were validated by assessing residual normality, and collinearity was assessed using variation inflation factor values. The sensitivity, specificity, positive predictive value, and negative predictive value of TcB for clinical decision-making were calculated. True positive and negative treatment decisions were based on the measured TSB and clinical guidelines for phototherapy [3,4]. The statistical software IBM SPSS Statistics version 23 (SPSS, Chicago, IL) was used for the statistical analysis of the data.

## Results

A total of 263 paired TSB and TcB samples from 95 patients were identified from eligible infants during the study period, which were further divided into four distinct GA-subgroups: 130 paired samples ≥ 37 weeks (60 term infants), 75 paired samples 34.0-36.6 weeks (21 late preterm infants), 27 paired samples 32.0-33.6 weeks (seven moderate preterm infants), and 31 paired samples < 32 weeks (seven very preterm infants). The TSB and TcB samples range in term (1.0-21.0 and 0.7-19.5 mg/dL), late preterm (0.5-15.9 and 1.1 -16.9 mg/dL), moderate preterm (0.8-12.3 and 3.4-16.1 mg/dL), and very preterm (3.8-10.2 and 5.4-13.8 mg/dL). Baseline demographic data and the paired sample characteristics are presented in Table 1. In addition to having a much smaller sample size, the < 32 weeks very preterm group consisted of a notably higher percentage of African-Americans (71%), less nothing per oral (NPO) (0%), and a higher need for phototherapy (84%).

**Table 1:** Baseline demographics and paired sample characteristics ^a^Other includes Asian (n=3), Native American (n=7), Biracial (n=1), and Other (n=22). n = number, TSB = Total serum bilirubin, TcB = Transcutaneous bilirubin

Characteristics	All Patients (n=95)	>/= 37 weeks (n=60)	34 - <37 weeks (n=21)	32 - <34 weeks (n=7)	<32 weeks (n=7)	p-value
Gestational Age (weeks)	36.6 ± 3.6	38.8 ± 1.3	35.2 ± 0.8	32.4 ± 0.5	26.9 ± 1.9	<0.001
Birth Weight (kg)	2.83 ± 0.85	3.27 ± 0.57	2.49 ± 0.42	1.91 ± 0.34	1.02 ± 0.29	<0.001
Male Sex: n (%)	51 (54)	36 (60)	9 (43)	3 (43)	3 (43)	0.482
Race: n (%)						0.006
Caucasian	43 (47)	27 (47)	11 (52)	3 (43)	2 (29)	
African-American	16 (17)	8 (14)	3 (14)	0 (0)	5 (71)	
Other^a^	33 (36)	22 (39)	7 (33)	4 (57)	0 (0)	
Vaginal Delivery: n (%)	54 (57)	38 (63)	8 (38)	4 (57)	4 (57)	0.266
Blood Type: n (%)						0.35
A	21 (31)	12 (29)	5 (46)	4 (57)	0 (0)	
B	8 (12)	5 (12)	0 (0)	1 (14)	2 (29)	
AB	2 (3.0)	2 (4.8)	0 (0)	0 (0)	0 (0)	
O	36 (54)	23 (55)	6 (54)	2 (29)	5 (71)	
Paired Sample Characteristics	All Patients (n=263)	>/= 37 weeks (n=130)	34 - <37 weeks (n=75)	32 - <34 weeks (n=27)	<32 weeks (n=31)	
Bias (TSB-TcB)	-0.86 ± 2.94	-0.55 ± 3.69	-0.75 ± 1.90	-1.18 ± 1.81	-2.12 ± 1.73	0.056
Feeding: n (%)						<0.001
Breast milk	83 (32)	39 (30)	17 (23)	7 (26)	20 (64)	
Formula	71 (27)	28 (22)	16 (21)	16 (59)	11 (36)	
Both	83 (32)	49 (38)	33 (44)	1 (3.7)	0 (0)	
Not Feeding	26 (9.9)	14 (11)	9 (12)	3 (11)	0 (0)	
Phototherapy: n (%)	101 (38)	39 (30)	25 (33)	11 (41)	26 (84)	<0.001
Postnatal Age (hours)	81 ± 42	80 ± 41	78 ± 41	92 ± 42	85 ± 45	0.482

**Figure 1:**
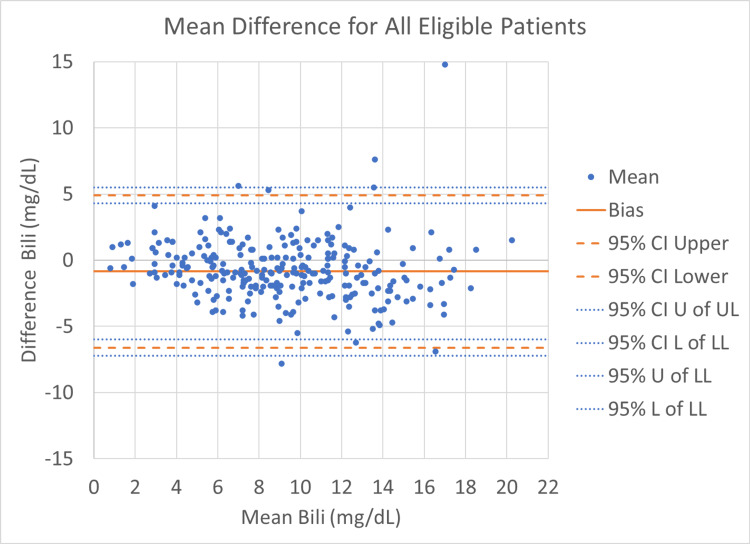
Transcutaneous bilirubin bias for the entire study population Bili = Bilirubin, CI = Confidence interval, UL = Upper limits, LL = Lower limits

The Bland-Altman agreement between TSB and TcB approximates TSB in term and preterm infants. The mean paired difference or bias across all the GA groups was -0.9 ± 2.9 mg/dL. As shown in Figure 1, 95% of our data fit within the limits of agreement, with only 5% out of agreement. We believe this is clinically negligible, and overall, our data suggest that on average, TcB overestimates the gold standard TSB by about 1-2 mg/dL.

**Figure 2:**
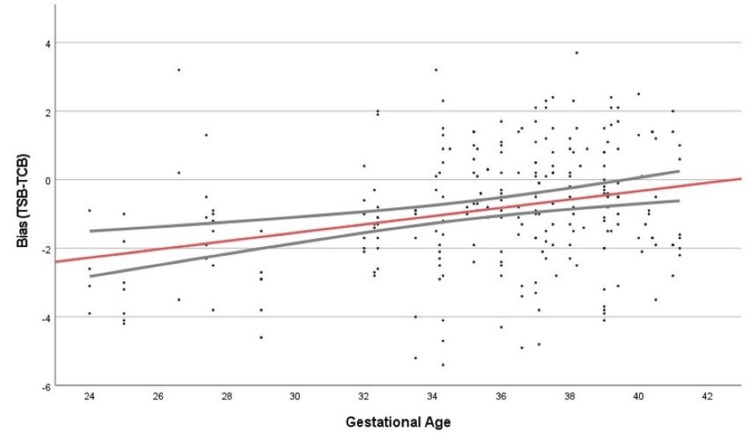
Model prediction with 95% confidence intervals The red line indicates the bias (TSB-TcB) across the gestational age range and includes the upper and lower 95% confidence intervals. TSB = Total serum bilirubin, TcB = Transcutaneous bilirubin

To further assess the effects of other variables on TcB bias, we performed multivariate linear regression on the difference between TcB and TSB with a stepwise entry for covariates that included GA (continuous) and race, feeding method, blood type, and presence of phototherapy (categorical) in Table 2. Twelve paired values were found to be outliers and removed prior to the model. The model was statistically significant (F=16.743, p<0.001) but with a poor overall fit (R2=0.091). Only GA (b=0.115, 95% CI 0.06-0.171, p<0.001) remained in the model (Table 2, Figure 2), and assumptions for multivariate linear regression (normality of residuals and heteroscedasticity) were met.

**Table 2:** Univariate and multivariable analysis of factors affecting TcB bias (R2=0.091, F=16.743, p<0.001) ^a^Includes Asian (n=9), Native American (n=20), Biracial (n=2), and Other (n=62). IV = Intravenous, n= Number

Parameter	Univariate		Multivariable	
	Mean or Rho (95% CI)	P value	Beta Coefficient (95% CI)	P-value
Gestational Age	0.139 (0.018, 0.256)	0.024	0.115 (0.060–0.171)	<0.001
Race				
Caucasian (n=108)	-1.2 (-1.79, -0.61)	0.003		
African-American (n=57)	-1.45 (-1.94, -0.97)			
Other^a^ (n=93)	0.02 (-0.44, 0.49)			
Blood Group				
A (n=54)	-0.27 (-1.05, 0.50)	0.179		
B (n=28)	-1.41 (-2.34, -0.485)			
AB (n=3)	-0.17 (-1.04, 0.71)			
O (n=96)	-0.91 (-1.3, -0.49)			
Feeding Method				
IV (n=83)	-0.60 (-1.2 to -0.048)	0.474		
Breastmilk (n=71)	-0.79 (-1.2, -0.37)			
Formula (n=83)	-1.1 (-2.2 to -0.66)			
Mixture (n=26)	-1.5 (-2.3 to -0.66)			
Phototherapy				
Yes (n=101)	-1.4 (-2.23, -0.60)	0.015		
No (n=162)	-0.51 (-0.78, -0.24)			

Finally, as shown in Table 3, we examined if there was a difference in agreement when making treatment decisions based on TSB and TcB values in neonates < 35 weeks GA. The TcB had a 92% sensitivity and 62% specificity for TSB values that would result in treatment with phototherapy. This resulted in a high negative predictive value (95%) for the test, which indicates it would be a safe screening tool for the need for a TSB measurement. However, the positive predictive value of the test was only 53%, which may result in additional TSB testing for false positives. Increasing the threshold for TSB screening by the bias in the TcB measurement for each GA would balance the sensitivity and specificity at 81% and 82%, respectively, and result in a negative predictive value of 90% and a positive predictive value of 68%.

**Table 3:** Intention to treat TSB vs. intention to treat TcB in preterm infants < 35 weeks GA TSB = Total serum bilirubin, TcB = Transcutaneous bilirubin, Y = Yes, N = No

		Treat TSB	
		Y	N
Treat TcB without bias correction	Y	24 (53%)	21 (47%)
	N	2 (5%)	35 (95%)
Treat TcB with bias correction	Y	21 (68%)	10 (32%)
	N	5 (10%)	47 (90%)

Cost-effectiveness

The hospital charge for TSB is $130.00/test. The cost of the Bilicheck device is $4,525.85 with no maintenance or replacement timeframe. Additional cost for TcB includes the cost of the sensor or calibration tip ($13.72/sensor) and Bili patch ($1.24/patch, but only if used while on phototherapy). In a conservative estimate based on our study data, if using TcB as the primary screening test can reduce one-third of TSB measurements over a six-month study period (263/3=66), the potential direct cost savings over a six-month period will be about $3,148.6 (the cost for 66 TSB testing = 66 x $130 = $8,580, cost for 66 TcB measurements = $5,431.37 ($4,525.85 + 66 x $13.72)).

## Discussion

Our results suggest TcB provides a reasonable estimate of TSB levels in term and preterm infants of various GA groups in NICU settings. The mean difference between TSB and TcB for our entire study population ranged from -0.6 to -2.1 mg/dL, which represents an overestimation of within 1-2 mg/dL with TcB. Though GA may affect the variability between TSB and TcB, especially in lower GA groups, in our small sample study, the decision to further test using TSB was not negatively affected by the bias but would result in additional TSB testing in about half of the cases. However, if the correction for GA bias were applied, there would be a minor drop (5%) in negative predictive value and a 15% increase in positive predictive value. If the TcB does not suggest treatment, then there is 90-95% certainty that TSB also will suggest ‘no’ treatment needed. However, if the TcB does suggest treatment, the possibility that TSB also will suggest treatment is only 53-68%. Our data suggest that a clinical practice change of making TcB the screening test of choice for the evaluation of jaundice in NICU settings can be justifiable due to its non-invasive nature and cost-reduction benefits. However, to use TcB as an alternative to TSB for treatment in preterm infants, a larger sample size study is needed for confirmation.

A 2013 systematic review on the reliability of TcB devices in preterm infants, < 37 weeks before initiating phototherapy, showed a narrow but inconsistent bias across 22 studies, ranging from an overestimation of about 3.27 mg/dL to an underestimation of about 1.31 mg/dL [10]. The results from the limited data of very preterm (< 32 weeks GA) infants included in this analysis suggested comparable diagnostic accuracy of TcB compared to the overall preterm population [10]. Many studies included in this systematic review provided multiple readings from each patient enrolled (similar to our study); however, a sensitivity analysis conducted as part of this systematic review using one data point per patient was similar to overall results with no significant heterogeneity [10]. Further studies that looked at the diagnostic accuracy of TcB in a preterm population showed similar results [12-17]. A recent prospective multicenter study of extreme preterm infants < 30 weeks gestation in California (n=141 with 755 paired TcB and TSB values) also observed a good correlation (R=0.786) between TcB and TSB within the TSB range of 0-12.6 mg/dl, supporting the use of TcB as a screening tool for the evaluation of jaundice in preterm infants [17]. Another recent prospective cohort study in preterm infants born at 23-34 weeks demonstrated an excellent area under the receiver operator characteristic curve (AUROC) for TcB (ranges from 0.85 to 0.96) for predicting TSB [20].

Our study also included infants on phototherapy and showed a positive agreement (acceptable bias) between patch TcB and TSB. Previous studies have noted variability between TcB and TSB in preterm infants undergoing phototherapy, but it was not significantly affecting management decisions in most studies [5,14,15]. Studies that assessed TcB on patched sternal skin vs. light-exposed skin of preterm infants undergoing phototherapy noted that patch TcB is more accurate and reliable to predict TSB [9,17,21,22]. Some studies found TcB tends to drift away from TSB levels when the TSB level exceeds 15 mg/dL even if measured in unexposed skin [23,24]. The use of such specific TcB cut-off levels may minimize the risk of missing high TSB levels while substantially reducing the need to obtain blood samples for TSB levels [25]. Our average postnatal age of the paired sample was > 72 hours, and the range of TSB tested in preterm infants was 0.8-13.8 mg/dL, including infants on phototherapy. Similar to Maisels et al. [4], our data also suggest that TcB is a reliable and practical initial screening method. A confirmatory TSB for treatment decisions should be obtained only if the TcB values are within 3 mg/dl from the treatment threshold.

Our study also suggests skin color, blood type, and feeding methods have no significant impact on the reliability of TcB. The finding related to skin color and TcB is consistent with earlier studies showing a good correlation between TcB and TSB from racially diverse populations [17,26]; however, some studies reported that, in Black African infants, TcB may overestimate TSB [23,27]. Our study showed that drift in TcB in a very preterm population may be attributed to the higher percentage of Black African infants in that group. Though this variability did not impact the management decisions, this needs to be confirmed in a larger sample size study. It also is possible that cutaneous maturation, bone depth, and light scattering that vary with gestational maturity and body location may explain the increasing variability seen in the very preterm GA group [28].

Our single-center NICU data suggest that routine use of TcB as the primary method to determine bilirubin levels can be potentially associated with cost savings, in addition to unmeasured potential nonfinancial savings in anxiety, pain, extra testing, etc. Similarly, a study that looked at the cost savings of TcB vs. TSB in a hospital and community setting has shown that the use of TcB as the primary screening test of choice is associated with about 70% reduction in blood draws for TSB with potential cost savings of $19,760 and $6,417, respectively, in a six-month period [29].

Our study has several limitations. This is a single-center retrospective chart review study, and we were not able to independently control the baseline patient demographics and sampling bias. Our sample size was limited to the very preterm group, and it had significant demographic differences compared to the more mature infant groups. However, these differences were not statistically significant predictors for TcB-TSB bias in the multivariate analysis. The maximum TSB level in preterm infants in our study was < 16 mg/dL, and study conclusions are applicable only to the specific TSB ranges reported. For cost analysis, insurance payment differences were not adjusted for - the net savings may be considerably less than reported in the results. In the first week of life, TSB is obtained often along with other routine labs in the NICU, thus reducing the need for TcB, which also will reduce the net cost savings associated with TcB adoption.

## Conclusions

Our study data suggest that TcB is a safe and potential cost-effective screening test for the evaluation of jaundice in preterm infants across GA groups in a real-world NICU setting. Our data, along with the increasingly robust evidence generated over the last few years, may contribute to a future meta-analysis on the use of TcB in preterm infants. The strategy of using TcB as the screening test of choice for neonatal jaundice will complement the overall strategy of NICUs seeking to improve clinical practice with more pain-free, cost-effective, and less time-consuming clinical management guidelines.
